# Trends, Social Determinants, and Lifestyle Factors Associated With Comorbidity of Diabetes and Kidney Diseases Among Chinese Adults Aged ≥ 45 Years

**DOI:** 10.1111/1753-0407.70142

**Published:** 2025-08-15

**Authors:** Guanghui Cui, Shaojie Li, Yu Qin, Yuqin Zhang, Kunpeng Hui, Mengfan Feng, Weiwei Li, Xuezhi Zhang

**Affiliations:** ^1^ Department of Integrated Traditional Chinese and Western Medicine Peking University First Hospital Beijing China; ^2^ Institute of Integrated Traditional Chinese and Western Medicine Peking University Beijing China; ^3^ School of Public Health Peking University Beijing China

**Keywords:** China, comorbidity, diabetes, kidney disease, lifestyles, social determinants

## Abstract

**Objectives:**

The dual burden of diabetes and kidney disease poses a growing public health challenge in aging populations, yet its longitudinal trends and modifiable determinants in China remain understudied. This study aims to track the trends in the prevalence of the comorbidity of diabetes and kidney disease in Chinese middle‐aged and older adults over a 10‐year period (2011–2020) and identify social determinants and lifestyle factors associated with this comorbidity.

**Methods:**

This study utilized data from five waves of the China Health and Retirement Longitudinal Study (CHARLS). Temporal trends and geographical distributions of the comorbidity of diabetes and kidney diseases were analyzed across survey years; disaggregated by sex, residence, and geographical region. Cox proportional hazards regression models were employed to explore the associations between SDOH burden, lifestyle factors, and the risk of comorbidity.

**Results:**

The prevalence of diabetes and kidney disease comorbidity exhibited a clear upward trend among Chinese adults aged ≥ 45 years, increasing from 0.60% in 2011 to 3.10% in 2020. Geographical analysis revealed significant regional disparities, with Northeast China having the highest prevalence throughout the decade (4.76% in 2020). High SDOH burden was associated with an increased risk of comorbidity (HR = 1.24, 95% CI = 1.01–1.52). Conversely, maintaining a good (HR = 0.38, 95% CI = 0.27–0.54) or moderate (HR = 0.74, 95% CI = 0.59–0.91) lifestyle was associated with a reduced risk.

**Conclusion:**

The rising prevalence and significant regional disparities of diabetes and kidney disease comorbidity underscore the urgent need for targeted public health interventions in China.

## Graphical abstract

This study reveals a fivefold increase in the prevalence of diabetes and kidney disease comorbidity among Chinese adults aged ≥ 45 years from 2011 to 2020. Significant regional disparities were identified, with the Northeast region bearing the highest burden in China. High social burden increased risk, while healthier lifestyles significantly reduced comorbidity.

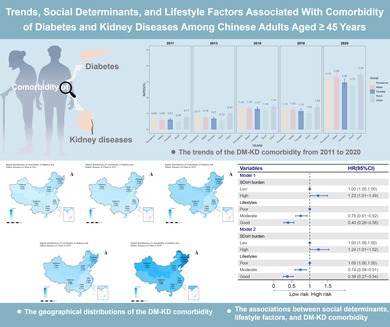

**Summary**

- The prevalence of diabetes and kidney disease comorbidity exhibited a clear upward trend among Chinese adults aged ≥ 45 years.
- Spatial analysis revealed significant regional disparities. High SDOH burden was associated with an increased risk of comorbidity. Conversely, maintaining a good or moderate lifestyle was associated with a reduced risk.
- These findings highlight urgent needs for region‐specific interventions and integrated strategies targeting SDOH and behavioral factors to alleviate the dual burden of diabetes and kidney disease.

## 1 Introduction

The escalating dual burden of diabetes mellitus (DM) and kidney disease (KD) represents a critical public health challenge, imposing profound morbidity risks on individuals and substantial economic strain on healthcare systems globally. Recent epidemiological projections underscore the urgency of this issue: the Global Diabetes Atlas (10th Edition) anticipates 783 million people living with diabetes by 2045, while chronic kidney disease (CKD) affected approximately 697 million individuals worldwide in 2017 [1, 2]. These conditions frequently coexist as comorbidities, driven by shared modifiable risk factors (e.g., obesity, hypertension, dyslipidemia) [3] and intertwined pathophysiological mechanisms involving metabolic dysregulation and renal dysfunction [4]. Emerging evidence reveals bidirectional relationships where KD may precede DM, coexist as a distinct entity, or exacerbate diabetic complications [5], amplifying clinical complexity and management challenges beyond traditional diabetic nephropathy paradigms.

This synergy elevates risks of catastrophic health outcomes, including cardiovascular mortality [6], progression to end‐stage renal disease [7], and all‐cause mortality [8, 9]. Despite these implications, current epidemiological understanding remains fragmented. Existing studies predominantly focus on clinical subpopulations—examining KD prevalence in diabetic cohorts [10, 11, 12, 13] or DM incidence in CKD patients [3, 7, 14]—leaving critical gaps in population‐level characterization. Specifically, longitudinal trends, geographic heterogeneity, and demographic patterns of DM‐KD comorbidity in community‐dwelling populations, particularly aging adults who bear disproportionate burdens, remain underexplored.

Beyond biological mechanisms, the etiology of DM‐KD comorbidity is shaped by multilevel determinants spanning social, environmental, and behavioral domains. Social determinants of health (SDOH)—encompassing socioeconomic status, education, and healthcare access—profoundly influence disease risks across the life course [15, 16]. Concurrently, lifestyle factors such as physical inactivity and poor dietary habits independently contribute to both DM and KD pathogenesis [17, 18]. However, three critical knowledge gaps persist: (1) limited integration of SDOH and lifestyle dimensions in comorbidity research; (2) insufficient attention to their longitudinal interplay in shaping disease trajectories; and (3) a paucity of evidence from rapidly transitioning economies like China, where urbanization and aging demographics intersect with stark rural–urban health disparities to create distinct risk profiles.

Leveraging nationally representative longitudinal data from the China Health and Retirement Longitudinal Study (CHARLS), this study addresses these gaps through three objectives: (1) track decade‐long epidemiological trends in DM‐KD comorbidity among Chinese adults aged ≥ 45 years, (2) map geographic disparities in comorbidity prevalence across diverse regions, and (3) quantify the joint contributions of SDOH and lifestyle factors to comorbidity risks. By synthesizing temporal, geographical, and socio‐behavioral insights, our findings aim to inform targeted prevention strategies under China's “Healthy Aging 2030” framework while offering a model for understanding multimorbidity dynamics in transitioning economies worldwide.

## 2 Materials and Methods

### 2.1 Study Design

**FIGURE 1:**
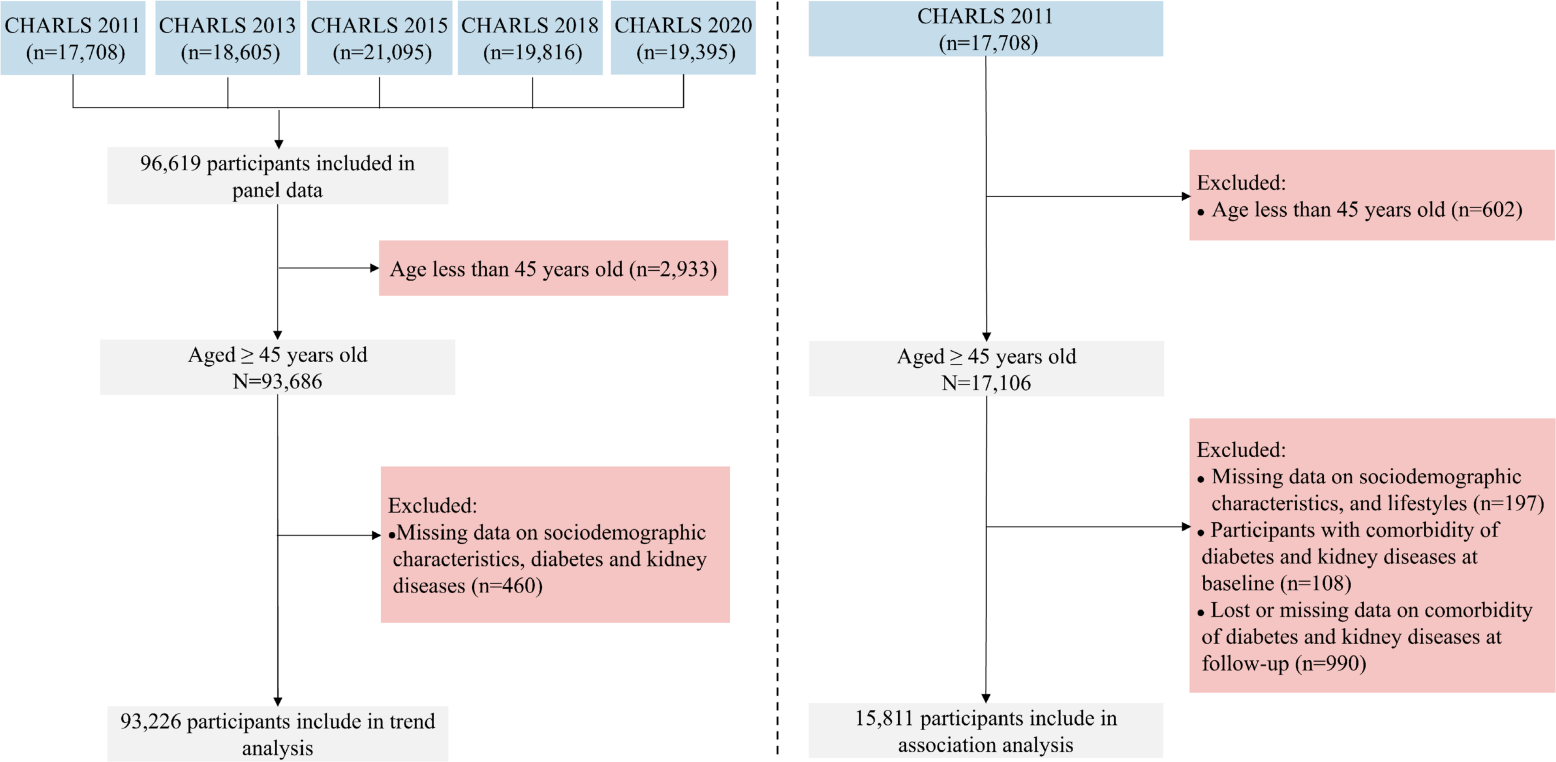
The select process of participants in this study.

This study utilized data from the CHARLS, a nationally representative longitudinal survey of Chinese adults aged 45 years and older. CHARLS aims to collect high‐quality data on the socioeconomic status, health, and family structure of middle‐aged and older populations in China [19]. For this study, data from five survey waves (2011, 2013, 2015, 2018, and 2020) were analyzed to assess temporal trends and geographical distribution characteristics of the DM‐KD comorbidity. In addition, we used a longitudinal analysis to explore the associations between SDOH, lifestyle factors, and the DM‐KD comorbidity. The 2011 survey served as the baseline, with subsequent waves (2013, 2015, 2018, and 2020) used for follow‐up analyses. A total of 17 708 participants were initially enrolled in the 2011 baseline survey. Participants were included in this study if they (1) were aged 45 years or older at baseline, (2) had complete data on diabetes and KD diagnoses at each wave, and (3) had information available on key covariates, including SDOH indicators and lifestyle factors. Participants were excluded if they (1) had missing data on diabetes or KD diagnoses, (2) had incomplete data for key covariates, or (3) were lost to follow‐up in all subsequent waves. After applying these inclusion and exclusion criteria, a total of 93 226 participants were included in the trend analysis, and 15 811 participants were retained for the survival analysis exploring the associations between SDOH, lifestyle, and comorbidity. Figure 1 shows the select process of participants in this study. Ethical approval for CHARLS was obtained from the Biomedical Ethics Review Committee of Peking University, and all participants provided written informed consent.

## 3 Measures

### 3.1 Comorbidity of Diabetes and Kidney Disease

Diabetes and KD are defined by the medical diagnosis in the CHARLS questionnaire: “have you ever been diagnosed with diabetes by a doctor?” and “have you ever been diagnosed with kidney disease (except for tumor or cancer)?” If participants answered yes to both of the above questions, they were considered to have a comorbidity of diabetes and KD.

### 3.2 Social Determinants

This study employed five domains of SDOH to examine their association with the comorbidity of diabetes and KD among older adults in China. These domains, adapted from the Healthy People 2030 framework, included economic stability, education access and quality, health care access and quality, neighborhood and built environment, and social and community context [20, 21].

Within the domain of economic stability, participants' income levels were assessed. Participants were classified as having low income if their income fell below the per capita disposable income for urban or rural areas, as defined in the 2011 China Statistical Yearbook. For education access and quality, the focus was on participants' educational attainment, categorized as having completed at least primary school or being illiterate. In the domain of health care access and quality, the presence of health insurance served as the indicator, measured as a binary variable denoting whether participants had coverage. The neighborhood and built environment domain was evaluated using the availability of basic living facilities, with a lack of tap water used as a proxy for inadequate living conditions, given the fundamental importance of water for daily life. Finally, the social and community context domain was represented by intergenerational communication, which measured the frequency of contact with children. Participants were considered to lack intergenerational communication if their interactions with children occurred less frequently than once per month.

To quantify the SDOH burden, we utilized the five indicators mentioned above, each categorized as either *unfavorable* (scored as 1) or *favorable* (scored as 0). The scores were then summed to create an aggregate SDOH burden score ranging from 0 to 5, with higher scores indicating a greater burden. In this study, an SDOH burden score of 3 or higher was categorized as high burden, while scores below 3 were categorized as low burden.

### 3.3 Lifestyles

Referring to a previous study [22, 23], this study constructs a Health Lifestyle Index that encompasses modifiable individual risk factors, including blood pressure, physical activity, body mass index (BMI), sleep duration, and smoking status. Blood pressure was measured by trained staff using standard sphygmomanometers, with the average of three measurements taken. Systolic blood pressure below 120 mmHg and diastolic blood pressure below 80 mmHg were considered indicative of well‐maintained blood pressure. Adequate physical activity was defined as engaging in vigorous exercise at least three times per week or moderate physical activity at least five times per week, with each session lasting no less than 30 min. BMI, calculated as weight divided by the square of height, was defined as healthy if it was less than 25 kg/m^2^ in this study. Sleep duration refers to the time spent asleep at night, with six or more hours considered good sleep. Additionally, smoking status was assessed by asking participants if they smoked, with non‐smoking being classified as good. Each of the five lifestyle factors was assigned a score of 0 (*poor*) or 1 (*good*), and these scores were summed to create a Health Lifestyle Score ranging from 0 to 5. In this study, lifestyles were categorized into three groups based on the score: poor (0–1 points), moderate (2–3 points), and good (4–5 points).

### 3.4 Statistical Analysis

Descriptive statistics were used to summarize the characteristics of the study sample and assess the temporal trends and geographical distributions of the DM‐KD comorbidity among middle‐aged and older adults in China. All prevalences were estimated using sampling weights from CHARLS. Temporal trends in prevalence rates from 2011 to 2020 were analyzed overall, as well as disaggregated by sex (males and females) and residence (urban and rural). Prevalence rates were calculated for each survey year, and trends over time were assessed both numerically and graphically. Geographical distributions of the comorbidity were analyzed across the six major geographical regions of China: North China, Northeast China, East China, South China, Southwest China, and Northwest China. Prevalence rates in each region were calculated for all survey years, and geographic trends were visualized to highlight geographical heterogeneity and regional disparities over time.

To investigate the association between SDOH, lifestyle factors, and the risk of diabetes and KD comorbidity, Cox proportional hazards regression models were employed. Both unadjusted and adjusted models were constructed to estimate hazard ratios (HRs) and 95% confidence intervals (CIs) for the associations. Covariates included in the adjusted models were age, sex, residence, and marital status. A sensitivity analysis was conducted by including baseline diabetes and KD status as covariates. Given that participants with baseline diabetes or KD might have an elevated risk of developing comorbidities due to underlying pathology, the inclusion of these variables helps validate the observed associations between SDOH burden, lifestyle quality, and comorbidity risk. All analyses used a significance threshold of *p* < 0.05. Statistical analyses were performed using STATA 17.0.

## 4 Results

### 4.1 Temporal Trends

**FIGURE 2:**
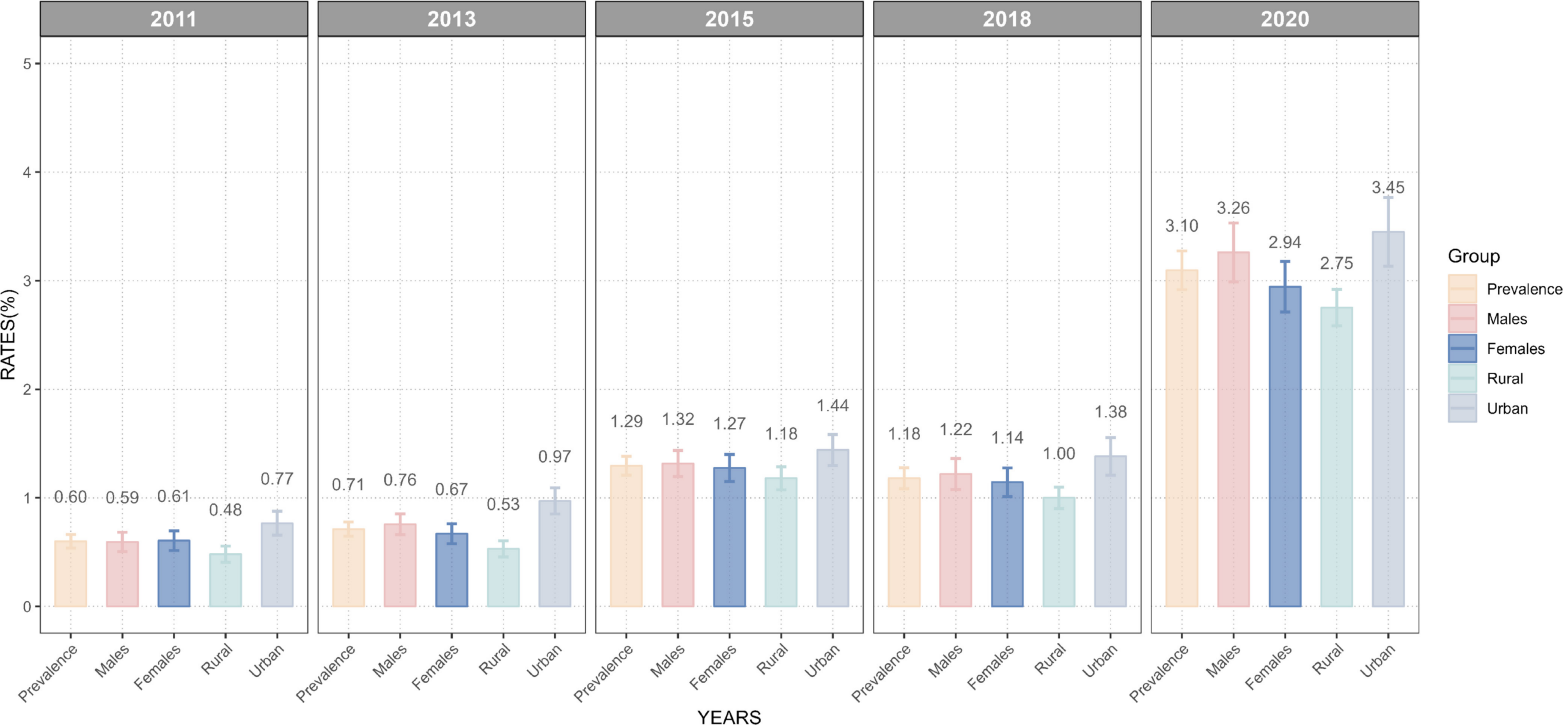
The trend of the DM‐KD comorbidity from 2011 to 2020.

The prevalence rates of DM‐KD comorbidity among middle‐aged and older adults in China exhibited a clear upward trend from 2011 to 2020 (Figure 2). Overall, the prevalence increased steadily, rising from 0.60% in 2011 to 3.10% in 2020. Among males, the prevalence increased from 0.59% in 2011 to 3.26% in 2020, marking a pronounced rise. Similarly, females saw an increase in prevalence from 0.61% in 2011 to 2.94% in 2020. When disaggregated by residence, the prevalence rates were consistently higher among urban areas compared to rural areas. In rural regions, the prevalence of diabetes and KDs rose from 0.48% in 2011 to 2.75% in 2020, reflecting a more than fivefold increase. In urban areas, the prevalence rates were consistently higher, starting at 0.77% in 2011 and surging to 3.45% in 2020. Figure 1 shows more details about the trend of this comorbidity from 2011 to 2020. Table S1 shows the sample characteristics used for trend and geographical distribution analysis.

### 4.2 Geographical Distributions

**FIGURE 3:**
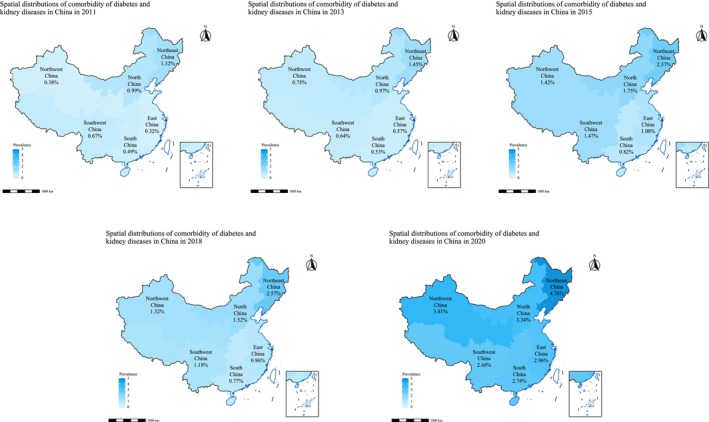
The geographical distributions of the DM‐KD comorbidity from 2011 to 2020.

The prevalence of DM‐KD comorbidity among middle‐aged and older adults in China exhibits significant geographical heterogeneity across the six geographical regions (Figure 3). Among the regions, Northeast China consistently displayed the highest prevalence rates throughout the decade. Starting at 1.32% in 2011, the prevalence rose steadily and peaked at 4.76% in 2020, representing the steepest increase among all regions. This highlights a pronounced and persistent burden in Northeast China over time. North China also demonstrated a significant upward trend. From a moderate prevalence of 0.99% in 2011, it rose sharply to 3.34% in 2020, making it one of the most affected regions by the end of the study period. Similarly, Northwest China exhibited a considerable rise, increasing from a relatively low prevalence of 0.38% in 2011 to 3.81% in 2020. East China, which initially had one of the lowest prevalence rates at 0.32% in 2011, experienced a notable increase, reaching 2.96% in 2020. While South China and Southwest China started with moderate rates of 0.49% and 0.67% in 2011, respectively, both regions exhibited steady growth over the years, with prevalence rates reaching 2.74% and 2.68% in 2020.

### 4.3 Social Determinants and Lifestyle Factors

**FIGURE 4:**
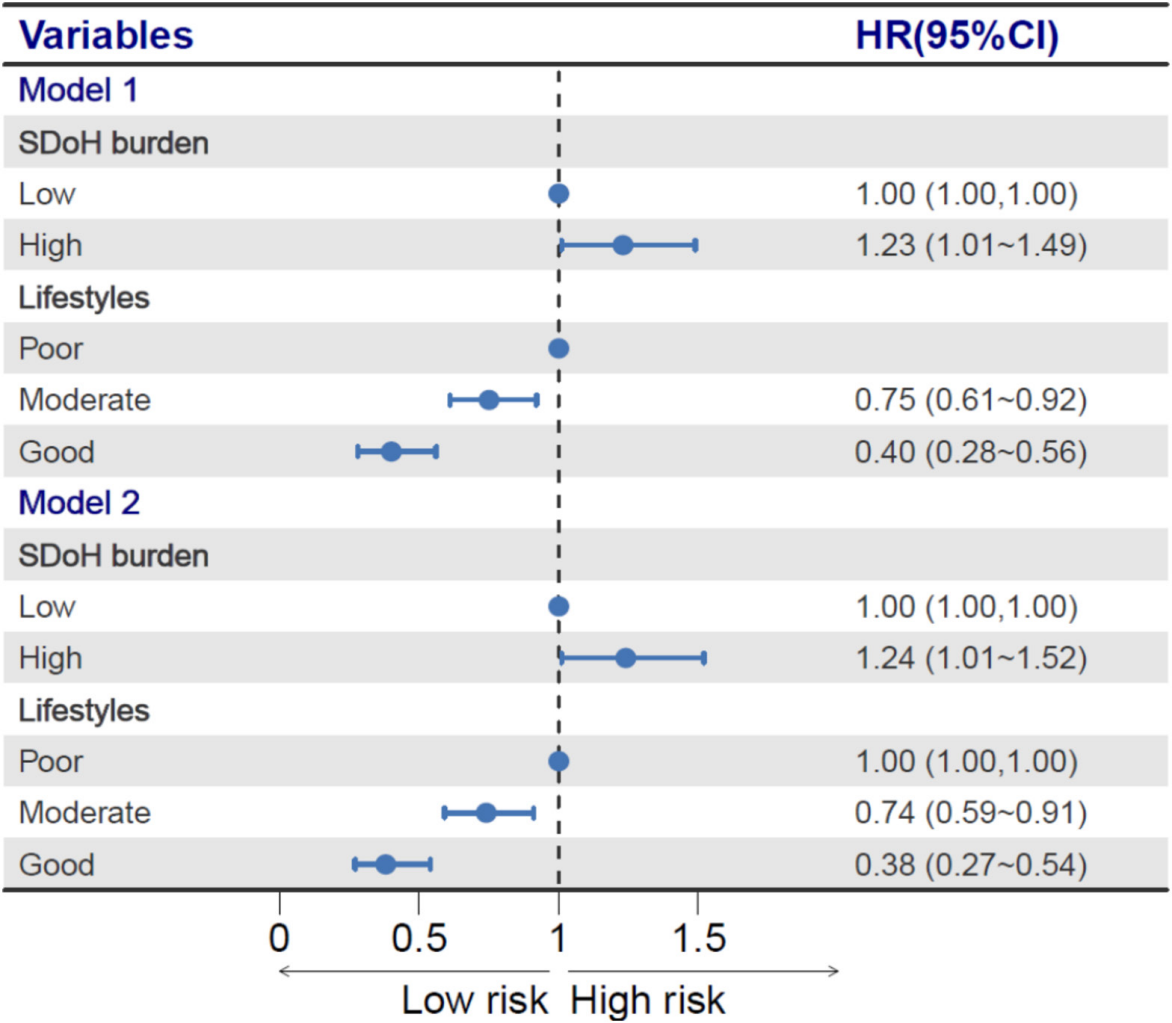
The forest plot of associations between social determinants, lifestyle factors, and DM‐KD comorbidity.

Table 1 displays the sample characteristics of longitudinal analysis. During a median follow‐up of 8.9 years, 480 incident DM‐KD comorbidity events were recorded. Table 2 and Figure 4 show the associations between social determinants, lifestyle factors, and the DM‐KD comorbidity. In the unadjusted and adjusted models, SDOH burden and lifestyles were associated with the DM‐KD comorbidity. After adjusting the covariates, high SDOH burden (HR = 1.24, 95% CI = 1.01–1.52) was associated with increased risk of the DM‐KD comorbidity, while maintaining a good (HR = 0.38, 95% CI = 0.27–0.54) and moderate (HR = 0.74, 95% CI = 0.59–0.91) lifestyle was associated with reduced risk of the DM‐KD comorbidity. The sensitivity analysis results of adding baseline diabetes and KD as covariates also found that high SDOH burden (HR = 1.22, 95% CI = 1.00–1.50), good (HR = 0.46, 95% CI = 0.33–0.66) and moderate (HR = 0.80, 95% CI = 0.65–0.99) lifestyles were associated with reduced risk of the DM‐KD comorbidity. We also assessed the interaction between SDOH burden and lifestyle factors on the risk of the DM‐KD comorbidity. However, no significant interaction effect was observed (*p* > 0.05). The subgroup analysis of SDOH burden showed that in the low (HR = 0.48, 95% CI = 0.31–0.72) and high (HR = 0.44, 95% CI = 0.23–0.85) burden groups, a good healthy lifestyle remained a significant protective factor for the DM‐KD comorbidity.

**TABLE 1:** Sample characteristics of longitudinal analysis.

Variables	Total	Incidence of DM‐KD comorbidity at follow‐up	
		No	Yes
Total sample	*N* = 15 811	*N* = 15 331	*N* = 480
Age	58.8 (9.5)	58.8 (9.5)	59.1 (8.3)
Sex			
Males	7673 (48.5%)	7438 (48.5%)	235 (49.0%)
Females	8138 (51.5%)	7893 (51.5%)	245 (51.0%)
Residence			
Rural	9644 (61.0%)	9367 (61.1%)	277 (57.7%)
Urban	6167 (39.0%)	5964 (38.9%)	203 (42.3%)
Marital status			
Married	13 860 (87.7%)	13 430 (87.6%)	430 (89.6%)
Unmarried	1951 (12.3%)	1901 (12.4%)	50 (10.4%)
Low income			
No	8247 (52.2%)	8010 (52.2%)	237 (49.4%)
Yes	7564 (47.8%)	7321 (47.8%)	243 (50.6%)
Educational attainment			
Primary school and above	11 502 (72.7%)	11 150 (72.7%)	352 (73.3%)
Illiterate	4309 (27.3%)	4181 (27.3%)	128 (26.7%)
Health insurance			
Yes	14 826 (93.8%)	14 367 (93.7%)	459 (95.6%)
No	985 (6.2%)	964 (6.3%)	21 (4.4%)
Tap water			
Yes	9668 (61.8%)	9400 (61.9%)	268 (56.4%)
No	5988 (38.2%)	5781 (38.1%)	207 (43.6%)
Intergenerational communication			
≥ Once a month	4752 (30.1%)	4615 (30.1%)	137 (28.5%)
< Once a month	11 059 (69.9%)	10 716 (69.9%)	343 (71.5%)
Blood pressure			
Poorly‐maintained	7370 (46.6%)	7113 (46.4%)	257 (53.5%)
Well‐maintained	8441 (53.4%)	8218 (53.6%)	223 (46.5%)
Physical activity			
Inadequate	14 806 (93.6%)	14 352 (93.6%)	454 (94.6%)
Adequate	1005 (6.4%)	979 (6.4%)	26 (5.4%)
BMI			
≥ 25 kg/m^2^	7156 (45.3%)	6879 (44.9%)	277 (57.7%)
< 25 kg/m^2^	8655 (54.7%)	8452 (55.1%)	203 (42.3%)
Sleep duration			
< 6 h	4261 (26.9%)	4093 (26.7%)	168 (35.0%)
≥ 6 h	11 550 (73.1%)	11 238 (73.3%)	312 (65.0%)
Smoking			
Yes	6370 (40.3%)	6187 (40.4%)	183 (38.1%)
No	9441 (59.7%)	9144 (59.6%)	297 (61.9%)
SDOH burden			
Low	11 294 (71.4%)	10 969 (71.5%)	325 (67.7%)
High	4517 (28.6%)	4362 (28.5%)	155 (32.3%)
Lifestyle			
Poor	2987 (18.9%)	2865 (18.7%)	122 (25.4%)
Moderate	10 036 (63.5%)	9724 (63.4%)	312 (65.0%)
Good	2788 (17.6%)	2742 (17.9%)	46 (9.6%)

**TABLE 2:** Associations between social determinants, lifestyle factors, and the DM‐KD comorbidity.

Variables	Events	Incidences (%)	Model 1		Model 2	
			HR (95% CI)	*p*	HR (95% CI)	*p*
SDOH burden						
Low	325/11 294	2.88	1 (Reference)	NA	1 (Reference)	NA
High	155/4517	3.43	1.23 (1.01–1.49)	0.037	1.24 (1.01–1.52)	0.038
Lifestyles						
Poor	122/2987	4.08	1 (Reference)	NA	1 (Reference)	NA
Moderate	312/10 036	3.11	0.75 (0.61–0.92)	0.007	0.74 (0.59–0.91)	0.005
Good	46/2788	1.65	0.40 (0.28–0.56)	< 0.001	0.38 (0.27–0.54)	< 0.001

*Note:* Model 1 is unadjusted; Model 2 adjusted age, sex, residence, and marital status.

## 5 Discussion

This study demonstrates a clear upward trend in the prevalence of comorbidity between diabetes and KDs among Chinese adults aged 45 years and older from 2011 to 2020. The prevalence of this comorbidity increased substantially from 0.60% in 2011 to 3.10% in 2020. The results indicate that both males and females experienced significant rises in prevalence, with males consistently showing higher rates. Furthermore, urban populations exhibited a higher prevalence of comorbidity compared to rural populations, although the latter experienced a more pronounced increase over time.

The observed trends in the prevalence of diabetes and KD comorbidity may be attributed to several factors. First, China's rapidly aging population has led to an increased burden of chronic diseases [24], as older adults are at higher risk for both diabetes and KDs. Previous studies have found that adequate access to healthcare has increased the life expectancy of older people in China [25], and correspondingly, their years of living with illness have also increased, which may be the reason for the increasing comorbidity rate among this group year by year. Additionally, lifestyle changes resulting from urbanization, such as increased sedentary behavior, poor diet, and higher rates of smoking [26], have likely exacerbated the prevalence of both conditions. These trends are particularly pronounced in urban areas, where urbanization has led to increased access to processed foods, a decline in physical activity, and increased obesity rates, all of which are key risk factors for diabetes and KD [3, 27]. The more than fivefold increase in the prevalence of comorbid diabetes and KD in rural areas may be attributed to several factors. First, with improvements in medical technology and diagnostic capabilities, there has been an increase in the identification and reporting of these chronic diseases in rural areas [28]. Second, as living standards improve, changes in diet and lifestyle in rural populations have contributed to a higher risk of developing these conditions [29, 30, 31]. Moreover, the rise in rural prevalence could also be influenced by limited access to healthcare and health awareness, which may delay diagnosis and management of these diseases. Previous research results also indirectly support our findings that the treatment rate of diabetes in rural areas is significantly lower than that in urban areas [32].

Notably, the dramatic increase in comorbidity rates of DM‐CKD between 2018 and 2020 (from 1.18% to 3.10%) may be largely attributable to the potential impact of the COVID‐19 pandemic. The COVID‐19 pandemic from late 2019 to 2020 may accelerate DM‐CKD risk through multiple pathways. First, during the pandemic, severe limitations in access to and utilization of healthcare services—including insulin and other medications, as well as blood glucose and renal function monitoring—led to disruptions in medical care for patients with preexisting diabetes [33]. This exacerbated poor disease control and comorbid complications. Secondly, approximately 19%–40% of COVID‐19‐related hospitalized patients have diabetes [34, 35, 36]. This suggests that a significant proportion of diabetic patients who had not undergone regular health assessments may have been newly identified with KD when they are evaluated for full treatment during admission to the hospital for COVID‐19. Furthermore, the kidneys are a primary target of SARS‐CoV‐2 infection. Cytokine storms, direct viral attack, and dysregulation of immune homeostasis can lead to acute kidney injury and other KDs, substantially increasing the comorbidity of diabetes and KD [37, 38]. Additionally, the Healthy China Initiative launched by the Chinese government in 2019 incorporated diabetes and its complications screening into national health targets. By 2020, this policy likely led to the identification of a large number of previously undiagnosed asymptomatic diabetes and KD cases.

The results of geographical distributions showed Northeast China emerged as the region with the highest and most rapidly increasing prevalence of diabetes–KD comorbidity, escalating from 1.32% in 2011 to 4.76% in 2020. This persistent burden aligns with existing studies documenting elevated rates of non‐communicable diseases in northeastern provinces [39, 40]. The region's dietary patterns are characterized by high salt (such as pickled vegetables) [41], coupled with reduced physical activity due to prolonged cold winters [42], which may contribute to metabolic dysregulation and renal impairment. Comparatively, North China's sharp rise in prevalence (0.99%–3.34%) mirrors trends observed in urbanizing areas, where rapid socioeconomic transitions have led to air pollution, unhealthy lifestyles, and increased obesity rates [43, 44, 45]—all established risk factors for diabetes and KD. The dramatic increase in Northwest China (0.38%–3.81%) is particularly striking, transitioning from the lowest to one of the highest prevalence regions. Limited healthcare infrastructure in rural Northwest China [46] may delay early diagnosis and management of diabetes, accelerating progression to kidney complications. Similarly, East China's rise from a low baseline (0.32%–2.96%) could be linked to urbanization‐driven lifestyle changes, despite its relatively advanced healthcare system. The moderate but steady growth in South and Southwest China (0.49%–2.74% and 0.67%–2.68%, respectively) suggests a slower but concerning trajectory, potentially mitigated by dietary habits rich in vegetables and lower obesity rates in these regions, as reported in prior studies [47, 48].

The findings of this study highlight the critical role of cumulative social disadvantage and modifiable lifestyle factors in shaping the risk of comorbid diabetes and KD among older adults in China. By conceptualizing SDOH as a composite burden rather than isolated factors, this analysis underscores the synergistic effects of structural inequities, while lifestyle behaviors emerged as powerful, independent protective pathways. The composite SDOH score—encompassing economic instability, limited education, inadequate healthcare access, poor neighborhood conditions, and weak social ties—reflects a multidimensional deprivation that amplifies vulnerability to chronic diseases. This aligns with the cumulative disadvantage theory [49], which posits that overlapping socioeconomic, environmental, and psychosocial stressors exacerbate biological wear‐and‐tear, accelerating disease onset and progression. While individual SDOH components were not analyzed separately, the aggregate burden score captures the lived reality of older adults navigating intersecting inequities. For instance, economic instability may limit access to diabetes medications, while inadequate education reduces health literacy, compounding poor disease management [50]. Similarly, lacking tap water—a proxy for neighborhood disadvantage—may reflect broader environmental risks (e.g., contaminated water, poor sanitation) that directly harm renal function [51]. The absence of intergenerational communication further isolates older adults, depriving them of caregiving support critical for chronic disease management [52]. These findings resonate with previous studies, which found the social deprivation index strongly associated with cardiometabolic multimorbidity [53].

In contrast to the SDOH burden, maintaining a good or moderate lifestyle demonstrated robust protective effects, even after adjusting for SDOH and other covariates. The Health Lifestyle Index, integrating blood pressure control, physical activity, healthy BMI, adequate sleep, and nonsmoking, underscores the importance of holistic behavioral modifications. Each component targets distinct pathways: blood pressure management reduces glomerular hypertension [54]; physical activity improves insulin sensitivity [55]; and smoking cessation mitigates vascular inflammation [56]. Notably, the protective association persisted in sensitivity analyses adjusting for baseline diabetes and KD, suggesting that lifestyle interventions remain beneficial even after disease onset. This aligns with trials like the Diabetes Prevention Program, where lifestyle changes prevented and delayed the progression of diabetes and CKD [57, 58]. However, the lack of interaction between SDOH burden and lifestyle factors indicates that lifestyle modifications confer protection regardless of social disadvantage. This finding suggests that the effects of SDOH burden and lifestyle factors on comorbidity risk operate independently, rather than synergistically. The subgroup analysis found that even in high‐burden settings, promoting healthy lifestyles may reduce the risk of comorbidity of diabetes and KD. Similarly, the protective role of lifestyles aligns with the study of China Kadoorie Biobank, which identified a healthy lifestyle as key drivers of reduced risk of type 2 diabetes [59].

This study provides critical epidemiological evidence for addressing the dual burden of diabetes and KD comorbidity in China's aging population. The observed 10‐fold increase in prevalence over a decade underscores the urgency of integrating comorbidity prevention into national chronic disease management strategies. From a public health perspective, our findings highlight three key intervention points: first, the persistent urban–rural disparity calls for targeted resource allocation to strengthen primary care capacity in rural areas. Second, the striking regional variations, with Northeast China demonstrating the highest burden, necessitate geographically tailored interventions that address local risk profiles—for instance, implementing salt‐reduction campaigns in northeastern provinces where preserved food consumption is prevalent. Third, the dual influence of SDOH burden and lifestyle factors reveals synergistic intervention opportunities: community‐based programs combining social support networks with lifestyle coaching could simultaneously mitigate structural disadvantages while promoting behavioral changes.

Several limitations should be considered when interpreting these findings. First, the reliance on self‐reported physician diagnoses may underestimate true prevalence due to low disease awareness in rural populations and potential recall bias. Second, the CHARLS dataset lacks detailed clinical parameters (e.g., HbA1c levels, urinary albumin–creatinine ratio) that could refine comorbidity characterization and severity stratification. Third, our SDOH assessment did not account for environmental exposures like air pollution and heavy metal contamination, which are emerging risk factors for both diabetes and KD in industrial regions. Fourth, the observational design precludes causal inferences; while lifestyle factors showed strong associations, residual confounding from unmeasured variables (such as medication adherence) may persist. Due to CHARLS data limitations, we are unable to cover more lifestyle factors (such as dietary patterns). Finally, the operational definition of KD encompassed non‐cancer diagnoses but could not differentiate between diabetic nephropathy and other renal pathologies.

## 6 Conclusion

The rising prevalence and significant regional disparities of diabetes and KD comorbidity underscore the urgent need for targeted public health interventions in China. Addressing SDOH burden and promoting healthier lifestyles could play a critical role in reducing the risk of this comorbidity among middle‐aged and older adults.

## Author Contributions

**Guanghui Cui and Shaojie Li:** writing – original draft, software, data curation, and conceptualization. **Yu Qin and Yuqin Zhang:** writing – review and editing, data curation. **Kunpeng Hui and Mengfan Feng:** writing – review and editing. **Weiwei Li and Xuezhi Zhang:** writing – review and editing, supervision, and funding acquisition.

## Ethics Statement

The studies involving human participants were thoroughly reviewed and received approval from the Ethics Committee of Peking University (IRB00001052‐11015).

## Consent

Written informed consent was obtained from all participants prior to their involvement in the study.

## Conflicts of Interest

The authors declare no conflicts of interest.

## Supporting information


**Table S1:** Characteristics used for trend and spatial distribution analysis

## Data Availability

The datasets utilized in this study can be accessed through the official China Health and Retirement Longitudinal Study (CHARLS) website (https://charls.pku.edu.cn/en/).
