# Supplementary material for: Trends, Social Determinants, and Lifestyle Factors Associated With Comorbidity of Diabetes and Kidney Diseases Among Chinese Adults Aged ≥ 45 Years
Source: J Diabetes. 2025 Aug 15;17(8):e70142. doi: 10.1111/1753-0407.70142 (PMC12355039; doi:10.1111/1753-0407.70142)
Supplement: Supplementary file 1 — Table S1: Characteristics used for trend and spatial distribution analysis [file JDB-17-e70142-s001.docx]

Trends and social determinants of comorbidity of diabetes and kidney diseases among Chinese older people: finding from five national representative surveys

Supplementary materials

Table S1 Characteristics used for trend and spatial distribution analysis

| Variables | 2011 | 2013 | 2015 | 2018 | 2020 |
| --- | --- | --- | --- | --- | --- |
|  | N=16930 | N=17967 | N=19718 | N=19454 | N=19157 |
| Age, mean (SD) | 59.1 (9.8) | 59.9 (9.9) | 60.2 (10.1) | 61.7 (10.2) | 61.8 (9.9) |
| Sex |  |  |  |  |  |
| Males | 8268 (48.8%) | 8730 (48.6%) | 9604 (48.7%) | 9283 (47.7%) | 9063 (47.3%) |
| Females | 8662 (51.2%) | 9237 (51.4%) | 10114 (51.3%) | 10171 (52.3%) | 10094 (52.7%) |
| Residence |  |  |  |  |  |
| Rural | 10079 (59.5%) | 10718 (59.7%) | 11729 (59.5%) | 11621 (59.7%) | 11474 (59.9%) |
| Urban | 6851 (40.5%) | 7249 (40.3%) | 7989 (40.5%) | 7833 (40.3%) | 7683 (40.1%) |
| Regions |  |  |  |  |  |
| North China | 2880 (17.0%) | 3040 (16.9%) | 3125 (15.8%) | 3208 (16.5%) | 3021 (15.8%) |
| Northeast China | 1268 (7.5%) | 1394 (7.8%) | 1433 (7.3%) | 1354 (7.0%) | 1185 (6.2%) |
| East China | 5107 (30.2%) | 5432 (30.2%) | 6197 (31.4%) | 5988 (30.8%) | 6021 (31.4%) |
| Central South China | 3584 (21.2%) | 3734 (20.8%) | 4035 (20.5%) | 3993 (20.5%) | 4100 (21.4%) |
| Southwest China | 2876 (17.0%) | 3112 (17.3%) | 3425 (17.4%) | 3421 (17.6%) | 3439 (18.0%) |
| Northwest China | 1215 (7.2%) | 1255 (7.0%) | 1503 (7.6%) | 1490 (7.7%) | 1391 (7.3%) |
